# A Single Amino Acid in Cucumber Mosaic Virus Determines Systemic Infection in Legumes: Species-Specific Differences in Key Residue Locations

**DOI:** 10.3390/ijms262311755

**Published:** 2025-12-04

**Authors:** Jisoo Park, Dongjoo Min, Gyeong Geun Min, Hangil Kim, Ju-Yeon Yoon, Rae-Dong Jeong, Jin-Sung Hong

**Affiliations:** 1 Agriculture and Life Science Research Institute, Kangwon National University, Chuncheon 24341, Republic of Korea; pjsbob12@naver.com; 2 Interdisciplinary Program in Smart Agriculture, Kangwon National University, Chuncheon 24341, Republic of Korea; perdues2@naver.com (D.M.); mean308@kangwon.ac.kr (G.G.M.); 3 Department of Forest Environment Protection, College of Forest and Environmental Sciences, Kangwon National University, Chuncheon 24341, Republic of Korea; imgksrlf@kangwon.ac.kr; 4 Department of Plant Protection and Quarantine, Jeonbuk National University, Jeonju 54896, Republic of Korea; jyyoon@jbnu.ac.kr; 5 Department of Applied Biology, Chonnam National University, Gwangju 61185, Republic of Korea; jraed2@jnu.ac.kr

**Keywords:** adzuki bean, *Vigna angularis*, legume, cucumber mosaic virus, 2a protein, site-directed mutagenesis

## Abstract

Adzuki bean (*Vigna angularis*), a major grain legume in Asia, is susceptible to infection by cucumber mosaic virus (CMV), which threatens crop productivity. Here, we characterized the CMV-Pa3 isolate from adzuki bean and investigated the role of specific amino acid residues in the viral 2a protein influencing systemic infection of legumes. Phylogenetic analysis demonstrated that CMV-Pa3 is genetically distinct from other legume-infecting isolates. Inoculation assays revealed that CMV-Pa3 causes systemic infection in adzuki bean, cowpea, soybean, and pea, whereas the control isolate CMV-Rs1 is restricted to inducing local necrotic lesions in cowpea and adzuki bean. Site-directed mutagenesis targeted two conserved amino acid positions (631 and 641) in the 2a protein of CMV-Rs1. Functional analysis showed that residue 631 (Tyr) facilitates systemic infection across all tested legumes, while alteration at position 641 (Ser) alone enables systemic infection in cowpea and pea. These findings identify amino acid determinants in the CMV 2a protein critical for overcoming host restrictions and mediating systemic infection in various leguminous species. This work offers new insights into the molecular mechanisms underlying CMV pathogenicity and host specificity.

## 1. Introduction

Adzuki bean (*Vigna angularis* Ohwi & Ohashi, synonym of *Phaseolus angularis*) is a crop belonging to the Fabaceae family and has been cultivated in Asia for a long time as an important grain [1,2]. *V. angularis* is susceptible to various plant diseases caused by pathogenic microorganisms [3,4], with viruses being particularly recognized as major pathogens that significantly affect the growth and yield of the plant [5,6]. Several plant viruses have been reported to infect *V. angularis*, including alfalfa mosaic virus (AMV) [7,8], azuki bean mosaic virus (AzMV) [5,7,9,10], bean common mosaic virus (BCMV) [5,9,11], blackeye cowpea mosaic virus (BlCMV) [5,9], cowpea mild mottle virus (CPMMV) [12], cucumber mosaic virus (CMV) [5,7,13], and soybean mosaic virus (SMV) [9,14], and most of these viruses are transmitted by insect vectors [15,16,17], such as aphids, causing severe impacts on the growth and yield of adzuki beans.

CMV is a plant virus belonging to the genus *Cucumovirus* in the family *Bromoviridae*, and it causes significant economic losses in various agriculturally important crops [18]. CMV has a broad host range of more than 1300 species across over 100 families and 500 genera, and many studies have investigated viral factors that determine its pathogenicity in different host plants [19,20,21]. The genome of CMV consists of three positive-sense single-stranded RNA segments, encoding a total of five proteins [20]. RNA1 encodes the 1a protein, which contains helicase and methyltransferase domains essential for viral replication. RNA2 encodes the 2a protein, an RNA-dependent RNA polymerase (RdRp) responsible for viral RNA replication [19,20]. Additionally, the 2b protein is translated from a subgenomic RNA and is involved in viral movement, pathogenicity, and suppression of the plant RNA silencing defense mechanism [22,23]. RNA3 encodes the movement protein (MP) and the coat protein (CP). MP facilitates cell-to-cell movement of the virus, and CP forms the viral particle, playing a critical role in virus protection and transmission [24].

CMV isolates have adapted to diverse hosts and are broadly classified into two subgroups (Subgroup I and II) based on serological properties and nucleotide and amino acid sequence homology [25,26,27]. Subgroup I (S-I) and Subgroup II (S-II) show distinct differences in symptom expression and host specificity [28,29]. CMV isolates from leguminous plants generally belong to the S-I subgroup. While most CMV isolates typically induce a hypersensitive response (HR) confined to inoculated leaves of leguminous hosts, certain isolates are able to overcome this host restriction and establish systemic infection [13]. Notably, conserved amino acid residues at positions 631 and 641 in the 2a protein, shared by CMV isolates from leguminous plants, have been identified as critical determinants for inducing systemic infection in cowpea (*V. unguiculata*) [30,31,32]. In CMV isolates that cause only local lesions in cowpea, mutations in these two amino acids result in systemic infection. Such examples highlight how a single amino acid mutation in the viral genome can greatly influence pathogenicity. Although all CMV proteins are reported to play crucial roles as pathogenicity determinants depending on the host plant, specific amino acid variations can significantly influence viral pathogenicity and infection patterns, as demonstrated by various studies [24,33]. These pathogenicity determinants are involved in processes such as suppression of viral replication, inhibition of cell-to-cell movement, and evasion of host plant defense mechanisms [24,34,35,36]. Research on CMV pathogenicity focuses on identifying host-specific pathogenicity determinants and understanding how these determinants contribute to viral spread and disease induction within the plant, ultimately aiding in the development of virus-resistant cultivars and improved control strategies.

Infectious clones of plant viruses consist of viral genomes inserted into plasmids or vectors as DNA. When these DNA clones are delivered to plant cells, the viral genome is expressed within the plant, leading to the formation of actual viral particles and subsequent infection. By generating mutations in viral genes through infectious clones, the effects of specific amino acid residues on viral pathogenicity, symptom development, and transmission ability can be studied [37,38,39,40,41].

In this study, we isolated and characterized a CMV-Pa3 from adzuki bean and examined its biological and molecular traits. We generated infectious clones and chimeric recombinants between CMV-Pa3 and CMV-Rs1 to investigate the effects of specific amino acid mutations in the 2a protein of CMV-Pa3 on systemic infection and symptom expression in leguminous plants. Unlike previous studies primarily confined to cowpea, our research extends the functional analysis of key residues (positions 631 and 641 in the 2a protein) to multiple leguminous host, including adzuki bean, common bean, and pea, thereby revealing broader host-dependent effects of these mutations. Moreover, we demonstrate that the 641st amino acid, previously considered less critical, can independently induce systemic infection in cowpea and pea, highlighting a previously unrecognized determinant of CMV virulence and host adaptation.

These findings advance the current understanding of CMV-legume interactions by uncovering novel molecular determinants involved in host adaptation and viral systemic movement. The results provide new insights into the interplay between viral replication machinery and host defense responses, contributing fundamental knowledge that may facilitate the development of resistant legume cultivars and more effective CMV management strategies.

## 2. Results

### 2.1. Genetic Characterization of CMV-Pa3

**Figure 1:**
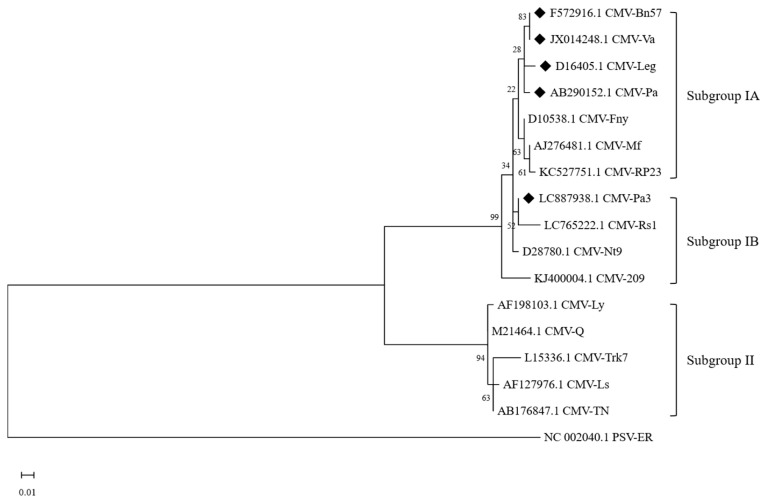
Phylogenetic tree of CMV-Pa3 and other CMV isolates based on their coat protein gene. The tree was reconstructed using the Maximum Likelihood method in MEGA11 with 1000 bootstrap replicates. Numbers at the nodes indicate the percentage occurrence of each node in the bootstrap resampling. Black diamonds represent the CMV isolates from leguminous plants.

We identified an infection of CMV in azuki bean plants and subsequently isolated the virus, which we have named CMV-Pa3. To understand its genetic characteristics and determine its relationship with other CMV isolates, we sequenced the coat protein gene of CMV-Pa3. The coat protein gene is a critical component of the virus genome, often used for phylogenetic analysis due to its role in viral structure and host interaction. Using the obtained sequence data, we constructed a phylogenetic tree to compare the nucleotide sequence of CMV-Pa3 with those of previously characterized CMV isolates. This analysis aimed to assess the evolutionary relationships and classify CMV-Pa3 within the established CMV subgroups. Our phylogenetic analysis demonstrated that CMV-Pa3 clusters closely with isolates classified under subgroup IB, indicating a high degree of genetic similarity and a close evolutionary relationship with subgroup IB (Figure 1). Among the subgroup IB isolates, CMV-Pa3 showed a close phylogenetic relationship to the Rs1 isolate, which was previously isolated from radish (*Raphanus sativus*) in Korea. Furthermore, while CMV-Pa3 belongs to subgroup IB, our analysis highlighted a notable contrast with other CMV isolates from leguminous plants. For instance, the ‘209’ isolate from wild soybean (*Glycine soja*) in Korea also falls within subgroup IB, showing some degree of genetic kinship with CMV-Pa3. However, other isolates from various leguminous hosts, such as ‘Bn57’ isolate from common bean (*Phaseolus vulgaris*), ‘Va’ and ‘Pa’ isolate from azuki bean, and ‘Legume’ isolate from cowpea (*Vigna unguiculata*), were classified within subgroup IA. These isolates display distinct genetic differences from the subgroup IB isolates, indicating divergent evolutionary paths.

### 2.2. CMV-Pa3 Infects Systemically in Various Leguminous Plants

**Figure 2:**
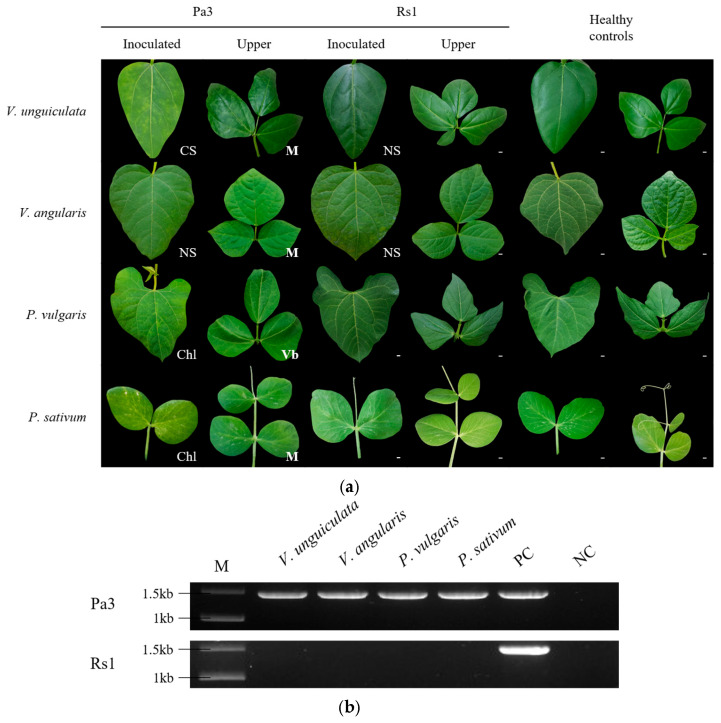
Host reactions and infectivity of CMV-Pa3 and CMV-Rs1 in leguminous plants. (**a**) Symptoms on inoculated and newly emerging leaves of cowpea, azuki bean, common bean, and pea caused by CMV-Pa3 and CMV-Rs1. Symptoms on inoculated leaves were observed at 3 dpi, and systemic leaves were observed at 7 dpi. (**b**) Results of RT-PCR analysis performed on the systemic leaves of each plant to confirm virus infection. PC and NC indicate positive and negative controls of the PCR assay, respectively. The white letters in the photos denote specific symptoms on each legume species. Chl, chlorosis; M, mosaic; NS, necrotic local lesion; Vb, vein banding.

We conducted inoculation experiments to test the infectivity and host responses of CMV-Pa3 in leguminous plants. Although CMV-Rs1 is not a legume isolate, it belongs to the same subgroup IB and was chosen as a control virus to compare host responses. We inoculated four species of leguminous plants—*V. unguiculata*, *V. angularis*, *P. vulgaris*, and *Pisum sativum*—with CMV-Pa3 and CMV-Rs1 using a mechanical inoculation method. After 3- and 7 days post inoculation (dpi), we observed symptoms on the inoculated and newly emerging leaves, respectively. CMV-Pa3, which was isolated from adzuki bean, caused systemic infection in all four legume species. In cowpea, adzuki bean, and pea, typical mosaic symptoms appeared on the systemic leaves. In *P. vulgaris*, vein banding was observed, characterized by yellowing and band-like discoloration along the veins. In contrast, CMV-Rs1 did not cause systemic infection in any of the four legume species (Figure 2a). To determine whether asymptomatic infections were present in the systemic leaves of host plants infected with CMV-Rs1, we performed RT-PCR to confirm the presence of the virus. As a result, CMV-Rs1 was not detected in the systemic leaves of any of the plants (Figure 2b).

We conducted a comparative analysis of CMV isolates previously known to be isolated from azuki bean to evaluate their host responses and ability to cause systemic infection in leguminous plants (Table 1). Interestingly, we found that among the azuki bean isolates, only CMV-Pa3 induced systemic infection in cowpea. This indicates that even CMV isolates derived from the same leguminous host do not necessarily induce systemic infection in all leguminous plants.

### 2.3. Comparison of Key Amino Acids in the CMV 2a Protein Related to Systemic Infection in Cowpea

Previous studies reported that systemic infection in cowpea is determined by two amino acids (631aa and 641aa) located within the 2a protein of CMV RNA2. Therefore, we compared the two amino acids in the 2a protein of CMV-Pa3 and other azuki bean isolates with those of CMV strains known to induce or not induce systemic infection in cowpea (Table 2).

Our analysis revealed that CMV-Pa3 shares the same amino acids—tyrosine (Tyr) at position 631 and serine (Ser) at position 641—with CMV-Leg, CMV-B, and CMV-P1, all of which are known to induce systemic infection in cowpea. Conversely, the adzuki bean isolates CMV-Va and CMV-RB, which do not cause systemic infection, possess the same amino acids as CMV-Fny and CMV-Y (phenylalanine (Phe) at position 631 and alanine (Ala) at position 641). Similarly, CMV-Rs1, an isolate from radish that does not induce systemic infection in cowpea, has the same amino acid sequence as CMV-Fny. Sequence comparisons of the nucleotide sequences of CMV isolates, obtained from the GenBank library, revealed a correlation between conserved nucleotide sequences at these two amino acid positions and the systemic infection phenotype.

### 2.4. A Chimeric Virus Containing Partial 2a Protein of CMV-Pa3 Induces Systemic Infection in Cowpea

**Figure 3:**
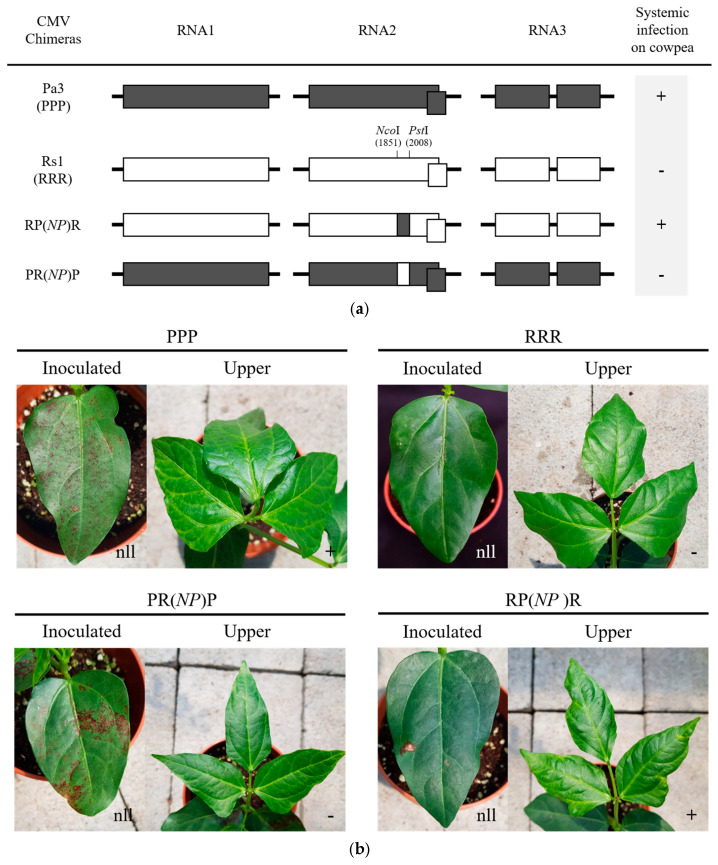
Identification of determinants for systemic infection in cowpeas through the construction of CMV chimeric viruses. (**a**) Genome schematic of CMV-Pa3 (PPP), CMV-Rs1 (RRR), and their chimeric viruses; (**b**) infectivity and symptoms in cowpeas. The gray area represents the genome of CMV-Pa3, and the white area represents the genome of CMV-Rs1. RNA transcribed from CMV cDNA clones was inoculated into the cotyledons of cowpea plants. Symptoms were observed on the inoculated leaves 3 days after inoculation and on the newly emerging leaves 10 days after inoculation. Systemic infection was indicated as + (positive) or − (negative). nll, necrotic local lesion.

We confirmed that the two amino acids in the 2a protein of CMV-Pa3 are identical to those in other legume isolates that induce systemic infection in cowpea. To determine if these two amino acids are crucial for systemic infection in cowpea, we constructed mutant viruses by substituting these amino acids using infectious clones of CMV-Pa3 and CMV-Rs1 (Figure 3a). To specifically assess the impact of these two amino acids on systemic infection, we used CMV-Rs1, which does not induce systemic infection, as the base strain and introduced substitutions at positions 631 and 641. We constructed a chimeric virus of CMV-Rs1 by employing the restriction enzyme sites containing both target amino acids (from nucleotide position 1851 to position 2008 in RNA2). This chimeric CMV-Rs1 RNA2, which incorporated both amino acids from CMV-Pa3, along with the RNA1 and RNA3 of CMV-Rs1, was named RP(NP)R. We obtained RNA transcripts via in vitro transcription, which were inoculated into cowpea, and their infectivity was compared with CMV-Pa3 (PPP) and CMV-Rs1 (RRR). While CMV-Rs1 (RRR) induces only necrotic lesions on the inoculated leaves of cowpea, CMV-Pa3 not only induces necrotic lesions on the inoculated leaves but also causes systemic infection (Figure 3b). The chimeric virus RP(NP)R, which includes a partial region of the 2a protein of CMV-Pa3, was confirmed to cause systemic infection in cowpea, similarly to CMV-Pa3. We also constructed a chimeric virus of CMV-Pa3 containing a portion of the 2a protein from CMV-Rs1 to compare its infectivity; however, this virus did not induce systemic infection. Therefore, the region from *Nco*I to *Pst*I within the 2a protein of CMV-Pa3 is crucial for determining systemic infection in cowpea.

### 2.5. Point Mutants at Amino Acid Positions 631 and 641 Induce Systemic Infection in Various Leguminous Plants

**Figure 4:**
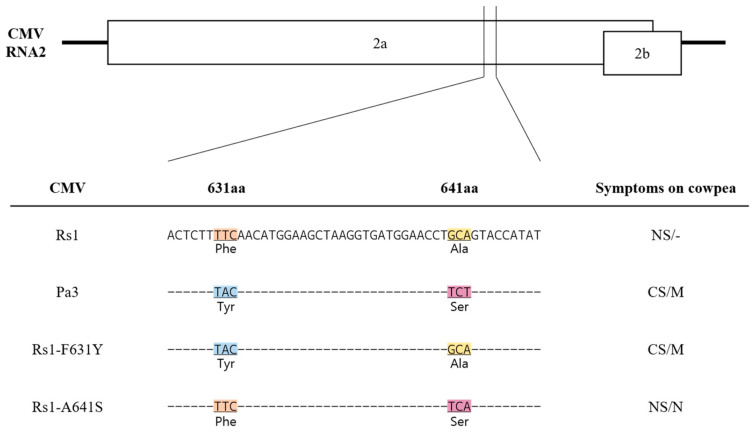
The 2a protein sequences on RNA2 of CMV-Rs1 and CMV-Pa3 and the sequence of point mutants generated in CMV-Rs1 that determine the systemic infection in cowpea. Identical amino acids are represented by the same color for clarity.

We previously demonstrated that specific regions of the 2a protein of CMV-Pa3 induce systemic infection in cowpeas, while CMV-Rs1 does not induce systemic infection. We also confirmed that CMV-Pa3, isolated from adzuki bean, shares the same amino acid sequence as CMV legume isolates known to induce systemic infection in cowpea. Sequence comparison revealed that the amino acids at positions 631 and 641 of the 2a protein of CMV-Pa3 are tyrosine (Tyr, Y) and Serine (Ser, S), respectively, whereas CMV-Rs1 has phenylalanine (Phe, F) and alanine (Ala, A) at these positions. To determine whether these specific amino acids are responsible for inducing systemic infection in cowpea and other leguminous plants, we constructed point mutants: Rs1-F631Y, which substitutes phenylalanine at position 631 to CMV-Rs1 with tyrosine, and Rs1-A641S, which substitutes alanine at position 641 of CMV-Rs1 with serine (Figure 4).

The infectivity of these point mutants (Rs1-F631Y and Rs1-A641S) was assessed by inoculating viral RNA transcripts obtained from an in vitro transcription system into tobacco plants (*Nicotiana benthamiana* and *N. tabacum* cv. Xanthi nc). The results showed that both mutant viruses systemically infected *N. benthamiana* and *N. tabacum* cv. Xanthi nc. Rs1-F631Y and Rs1-A641S induced severe mosaic symptoms with vein banding in *N. benthamiana* and mosaic symptoms in *N. tabacum* cv. Xanthi nc (Figure S1).

**Figure 5:**
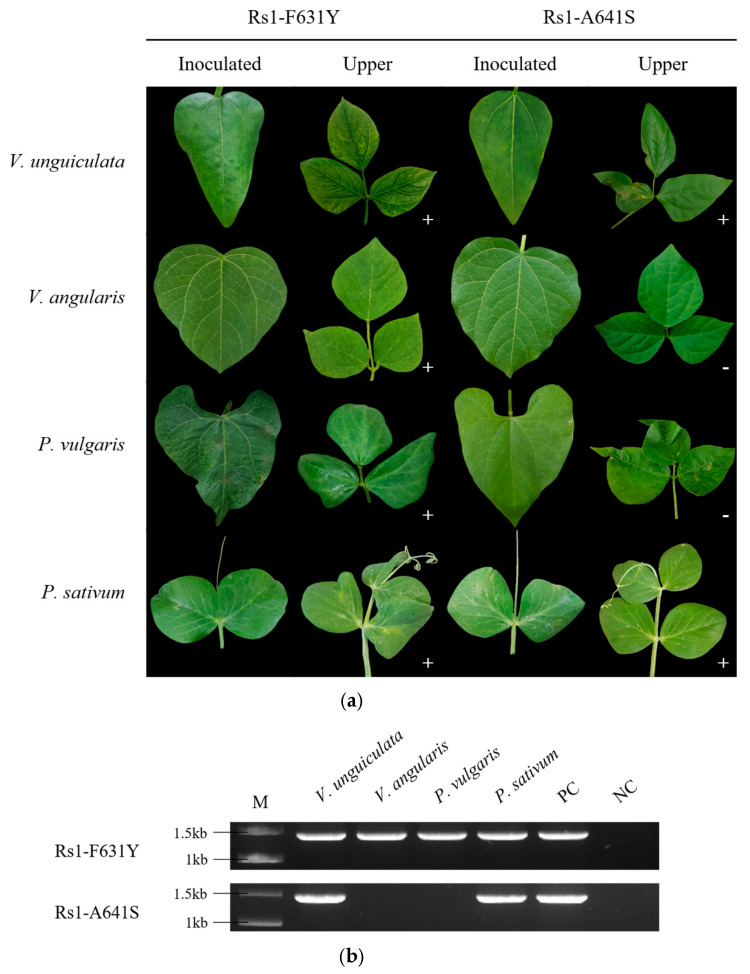
Systemic infection and the symptoms in leguminous plants inoculated with the CMV RNA2 point mutants (Rs1-F631Y and Rs1-A641S). (**a**) Symptoms induced by Rs1-F631Y and Rs1-A641S on inoculated and systemic leaves of four leguminous plants; (**b**) RT-PCR results. The +/− symbols in the photograph indicate RT-PCR positive (+) and negative (−) results.

Furthermore, the point mutants Rs1-F631Y and Rs1-A641S were inoculated onto adzuki bean, common bean, and pea (Figure 5). The single mutant Rs1-F631Y, corresponding to the 631st amino acid, was confirmed to induce systemic infection with mosaic symptoms in all tested plants, including adzuki bean, common bean, and pea. In contrast, the single mutant Rs1-A641S, at position 641, induced systemic infection only in cowpea and pea (Figure 5a). Virus infection in asymptomatic systemic leaves was confirmed by RT-PCR for all inoculated plants (Figure 5b). Additionally, we performed RT-PCR on systemic leaves and cloned the amplified DNA fragments into a TA cloning vector. Sequencing of the cloned fragments confirmed that the nucleotide sequences corresponded to the intended mutant type.

These results suggest that the amino acids at positions 631 and 641 of the 2a protein are associated with changes in infectivity across various leguminous plants. Moreover, the 631st amino acid plays a crucial role in determining systemic infection across a broader range of hosts, while the 641st amino acid, previously reported to have a relatively lesser role, is also shown to be a determinant of systemic infection in cowpea and pea.

## 3. Discussion

In this study, we identified and characterized a CMV isolate from adzuki bean, designated CMV-Pa3, which induces systemic infection in various leguminous plants. Our study confirms the evolutionary divergence between CMV isolates from leguminous plants. Notably, all previously reported CMV isolates from leguminous plants belong to subgroup IA [13]. Phylogenetic analysis based on the coat protein gene placed CMV-Pa3 in subgroup IB, closely related to the Rs1 isolate from radish. Interestingly, while both CMV-Pa3 and Rs1 belong to subgroup IB, their infection behavior in leguminous hosts differs significantly. This observation suggests that while CMV isolates within a given subgroup may share a common evolutionary origin, minor differences at specific amino acid positions can result in substantial changes in host range and pathogenicity. By comparing it to CMV-Rs1, a related isolate from the same subgroup, we sought to determine the viral factors that drive systemic infection in leguminous hosts.

One of the key findings of our study is that CMV-Pa3, unlike CMV-Rs1, causes systemic infection in four leguminous plants: cowpea, adzuki bean, common bean, and pea. This contrasts with CMV-Rs1, which induces only localized necrotic lesions in these hosts. Previous studies have shown that the 631st and 641st amino acids of the 2a protein in CMV RNA2 are critical determinants of systemic infection in cowpea, and our study expands this finding to other leguminous hosts, including adzuki bean and pea.

Building on previous findings that identified RNA2 of CMV as a key factor for systemic infection in cowpea, we targeted RNA2 to investigate its effect on the infectivity of CMV-Pa3. However, it has also been suggested that RNA3 of CMV influences hypersensitive response (HR) and systemic infection depending on the cowpea cultivar. To address this possibility and rule out the involvement of other RNA segments, we conducted preliminary experiments involving reassortant viruses (Figure S2). The results demonstrated that only the viruses containing RNA2 from CMV-Pa3 were capable of inducing systemic infection in cowpea. This reinforces our conclusion that the determinant for systemic infection in CMV-Pa3 is localized within RNA2.

The construction of chimeric viruses and point mutants allowed us to suggest the involvement of the 631st and 641st amino acids in systemic infection. Our results demonstrated that both 631aa (Tyr) and 641aa (Ser) in CMV-Pa3 are important for systemic infection in various leguminous plants, while a single amino acid at each position alone was sufficient to induce systemic infection. In particular, we discovered that the amino acids at positions 631 and 641 alone determine systemic infection in pea, a host that had not been previously tested. This indicates that these two residues serve as determinants of systemic infection in a wider range of hosts, not only in cowpea. These findings challenge the previously held view that the 641st amino acid plays a less significant role in systemic infection of leguminous plants.

The 2a protein of CMV plays a vital role in the virus’s replication mechanism, functioning together with the 1a protein within the viral replication complex [19,20]. Therefore, mutations in the amino acids within the 2a protein could potentially affect the enzymatic activity responsible for viral replication, which may in turn influence the virus’s capacity for systemic infection. Interestingly, despite our focus on the critical amino acids at positions 631 and 641, previous studies involving mutant viruses with substitutions at these positions found no significant difference in viral concentration when compared to the wild type [42]. This suggests that the inhibition of systemic infection due to mutations in the 2a protein is likely not linked to changes in replication efficiency.

The broader implications of our findings are twofold. First, our study highlights the importance of understanding the molecular determinants of systemic infection, not only for advancing our knowledge of plant–virus interactions but also for the development of effective control strategies. The identification of key amino acids responsible for systemic infection provides potential targets for breeding virus-resistant leguminous crops. Second, the use of infectious clones and point mutants in this study demonstrates the power of reverse genetics approaches in dissecting the roles of specific viral proteins in host adaptation and pathogenicity. Such approaches are critical for unraveling the complex interactions between viruses and their host, particularly in economically important crops like legumes.

## 4. Materials and Methods

### 4.1. Virus Source and Host Reaction Tests

Cucumber mosaic virus (CMV) was identified from an azuki bean plant exhibiting typical mosaic symptoms of viral infection. The CMV-Pa3 isolate, propagated in *Nicotiana tabacum* cv. Xanthi nc, was mechanically inoculated into four legumious plants to investigate its host reactions. The tested species included *Vigna unguiculata*, *V. angularis*, *P. vulgaris*, and *P. sativum*. The mechanical inoculation was performed by dusting carborundum powder onto the leaf surface and gently rubbing the sap, prepared by grinding infected leaf tissue in 0.01 M phosphate buffer (pH 7.2), onto the leaves by hand. For virus propagation, *N. tabacum* plants were inoculated at the three- to four-leaf stage, whereas Fabaceae plants were inoculated onto the first true leaves (bifoliate leaves) 5–7 days after sowing. All inoculations were conducted in three independent replicates, using CMV-Rs1 as a control isolate. Although minor variations were observed in symptom severity (such as the number of local lesions on inoculated leaves), all tested plants exhibited consistent symptom patterns. Plants were maintained in a controlled growth chamber at 25 °C under a 16 h light and 8 h dark photoperiod.

### 4.2. Total RNA Extraction and RT-PCR

Total nucleic acids containing viral RNA were extracted using the Plant RNA Prep kit (Biocube System, Suwon, Republic of Korea). Reverse transcription (RT) reaction was carried out using M-MLV reverse transcriptase (Promega, Madison, WI, USA) in reaction volume of 20 μL at 42 °C for 60 min and 93 °C for 3 min. RT mixtures contain 50–100 ng viral RNA, 50 mM Tris-HCl (pH 8.3), 75 mM KCl, 3 mM MgCl_2_, 10 mM DTT, 2 mM of each dNTP, 0.2 pmol reverse primer, 1 units RiboLock RNase inhibitor (Thermo Fisher Scientific, Waltham, MA, USA) and 100 units M-MLV reverse transcriptase. The target regions for PCR verification of virus infection correspond to nucleotides from position 1580 of the RNA2 2a protein gene to the 3′ terminus. These primers, named forward primer CMV-R2-1580-F and reverse primer SSVK3-SphI-R, were designed to detect nucleotides encoding the amino acids at positions 631 and 641. Primer sequence information is provided in Table S1. The target regions were amplified by polymerase chain reaction (PCR) from RT products by thermal cycler (Bio-Rad, Hercules, CA, USA). PCR was performed with total volume of 50 μL containing 2 μL of cDNA, 10 mM Tris-HCl (pH 8.3), 50 mM KCl, 1.5 mM MgCl_2_, 2.5 mM of each dNTP, 0.5 pmol forward primer, 0.5 pmol reverse primer, 10 units Taq DNA polymerase (Takara Bio, Shiga, Japan). PCR condition was as follows: pre-denaturation step (94 °C for 5 min), second step with 35 cycles (denaturation at 94 °C for 30 s, annealing at 50 °C for 30 s, extension at 72 °C for 1 min), and final extension step (72 °C for 5 min). The final amplified products were examined by 1.0% agarose gel electrophoresis. The amplified PCR product was cloned into the pGEM T-easy vector system (Promega, Madison, WI, USA) following the manufacturer’s instructions, and sequencing was performed by Macrogen (Seoul, Korea) using the vector’s T7 and SP6 promoter primers.

### 4.3. Phylogenetic Analysis

The nucleotide sequences of the coat protein gene and their encoded amino acid sequences were aligned in MEGA software (version 11). The aligned nucleotides of genomic sequences of 17 isolates were used for phylogenetic analysis. The phylogenetic analysis was carried out using the Maximum likelihood 1000 bootstrap replicates were used for phylogenetic analysis. The genomic sequence of the Peanut stunt virus (PSV) ER isolate was used as an outgroup taxon. The nucleotide sequences and deduced amino acid sequences of other representative CMV strains were obtained from the GenBank database. The GenBank Accession No. of the reference isolates: 209 (KJ400004.1), Bn57 (HF572916.1), Fny (D10538.1), Leg (D16405.1), LS (AF127976.1), LY (AF198103.1), Mf (AJ276481.1), NT9 (D28780.1), Pa (AB290152.1), Pa3 (LC887938.1), Q (M21464.1), RP23 (KC527751.1), Rs1 (LC765222.1), TN (AB176847.1), Trk7 (L15336.1), Va (JX014248.1), PSV-ER (NC 002040.1).

### 4.4. Construction of Infectious cDNA Clones

The primers used to construct the full-length cDNA clones of CMV Pa3 and for genome sequencing are shown in Table S1. cDNA of RNA1, RNA2, and RNA3 were synthesized by ‘SSVK3-*Sph*I-R’ primer and the segments of the CMV genome were amplified by respective primer pairs. RT-PCR was conducted under the following conditions: pre-denaturation step (94 °C for 5 min), second step with 35 cycles (denaturation at 94 °C for 30 s, annealing at 50 °C for 30 s, extension at 72 °C for 2 min), and final extension step (72 °C for 5 min). The amplified PCR product was cloned into the pGEM T-easy vector system (Promega, Madison, WI, USA) according to the manufacturer’s manual and sequenced by Macrogen (Seoul, Korea) using the T7 and SP6 promoter sequences present within the vector.

### 4.5. CMV Chimera and Point Mutagenesis

To construct chimeric RNA2 genomes between CMV-Rs1 and CMV-Pa3, we targeted nucleotide positions 1978 and 2007 of the 2a protein coding region, which correspond to amino acid residues 631 and 641, respectively. The full-length cDNA clones of CMV-Rs1 were used from a previous study [41]. The *Nco*I restriction site at position 1851 and the *Pst*I site at position 2008 on CMV-Rs1 RNA2 were utilized to facilitate the exchange. A region spanning nucleotides 1851 to 2008 of CMV-Pa3 RNA2 was amplified using specific primers (*Nco*I-Pa3-1851-F and *Pst*I-Pa3-2008-R) containing *Nco*I and *Pst*I recognition sequences. The resulting ~158 bp PCR amplicon was digested with *Nco*I and *Pst*I and ligated into the infectious cDNA clone of CMV-Rs1 RNA2 that had been digested with the same enzymes.

To generate point mutants at the target positions, two separate sets of primers were designed to introduce single-nucleotide substitutions. For site-directed mutagenesis of CMV-Rs1 RNA2, primer ‘G1978P-R’ and ‘G2007P-R’ were used in combination with primer ‘CMV-I-RNA2-T7-*Bam*HI-F’ to amplify the left fragment of CMV RNA2, while primer pairs ‘G1978P-F’ and ‘G2007P-F’ were used with primer ‘SSVK3-*Sph*I-R’ to amplify the right fragment. The sequence information of the primers used in the experiment is provided in Table S1. The mutated fragments were then assembled into full-length RNA2 by fusion PCR. Fusion PCR was performed under the same conditions as described above, using 1 µL of each cDNA fragment in the reaction mixture.

### 4.6. In Vitro Transcription

The sequences of forward primer used for cloning of CMV RNA1, 2 and 3 full genomes include restriction enzyme site (*Bam*HI), 19 nucleotides of 5′ terminal region of CMVs and T7 promoter (Table S1). T7 promoter was inserted to initiate transcription. The cDNA clones of CMV-Rs1 RNA1, CMV-Rs1 RNA3 and RNA2 point mutants (Rs1-F631Y and Rs1-A641S) were linearized by *Sph*I, existing on the end of 3′ terminal. The digested cDNA infectious clones were transcribed using the MEGAscript™ T7 kit (Invitrogen, Carlsbad, CA, USA) in the presence of 40 mM m7G(5′)ppp(5′)G RNA Cap Structure analog (New England Biolabs, Ipswich, MA, USA). The yield of the transcripts in the reaction mixture was analyzed on a 1.2% agarose gel. RNA transcripts (the mixture of RNA1, RNA2 mutants and RNA3 transcripts) were used to mechanically inoculate the leaves of *N. benthamiana* and *N. tabacum* cv. Xanthi nc. The ratios of CMV genomic RNA segments were maintained at 1:1:1 (RNA1:RNA2:RNA3). A final total RNA concentration of 1 μg was used for inoculation. The selection of the RNA concentration and inoculation method was based on the protocols described in the following study [43]. Quantification of the RNA was performed using a Colibri+ Microvolume Spectrophotometer (Berthold Technologies Bioanalytics, Bad Wildbad, Germany), ensuring measurement of RNA concentrations prior to inoculation.

## 5. Conclusions

The identification of key determinants in the 2a protein of CMV-Pa3, particularly the 631st and 641st amino acids, represents a significant advancement in our understanding of CMV pathogenicity. Our findings not only contribute to the broader knowledge of CMV–host interactions but also have practical implications for the management of CMV in leguminous crops. Future studies should explore the broader host range of CMV-Pa3 and investigate whether similar amino acid determinants exist in other CMV isolates that infect different plant families. Additionally, understanding the interaction between these viral determinants and host factors could pave the way for novel approaches to crop protection.

## Figures and Tables

**Table 1:** Infectivity of CMV isolates from azuki bean in leguminous plants.

Plants	Systemic Infection ^1^					
	Pa3	Pa ^2^	CM-19 ^3^	Va ^4^	RB ^5^	Rs1 ^6^
Azuki bean (*Vigna angularis*)	+	+	+	+	+	−
Broad bean (*Vicia faba*)	nt	nt	−	−	−	nt
Common bean (*Phaseolus vulgaris*)	+	nt	−	−	−	−
Cowpea (*V. unguiculata*)	+	−	−	−	−	−
Mung bean (*V. radiate*)	nt	nt	−	+	nt	nt
Pea (*Pisum sativum*)	+	nt	+	−	−	−

^1^ +; infected systemically; −, not infected; nt, not tested. ^2^ CMV ‘Pa’ isolated from *Phaseolus angularis* [7,10]. ^3^ CMV ‘CM-19’ isolated from *Phaseolus angularis* [5]. ^4^ CMV ‘Va’ isolated from *Vigna angularis* var. niponensis [13]. ^5^ CMV ‘RB’ isolated from *Vigna angularis* [32]. ^6^ CMV ‘Rs1’ isolated from *Raphanus sativus* and used as control virus in this study.

**Table 2:** Comparison of nucleotide and amino acid sequences of the 2a protein on RNA2 among CMV isolates.

Origin Host	CMV Isolate	Systemic Infection on Cowpea	Nucleotide (Amino Acid) at Position Number	
			1978 (631)	2007 (641)
Leguminous plant				
Azuki bean	Pa3	+	UAC (Tyr)	UCU (Ser)
	Va	−	UUC (Phe)	GCA (Ala)
	RB	−	UUC (Phe)	GCA (Ala)
Cowpea	Leg	+	UAC (Tyr)	UCA (Ser)
Common bean	B	+	UAC (Tyr)	UCA (Ser)
Pea	P1	+	UAC (Tyr)	UCA (Ser)
Non-leguminous plant				
Zucchini	Fny	−	UUC (Phe)	GCC (Ala)
Tobacco	Y	−	UUC (Phe)	GCA (Ala)
Radish	Rs1	−	UUC (Phe)	GCA (Ala)

## Data Availability

The complete genome sequences of CMV-Pa3 and CMV-Rs1 used in this study have been deposited in the NCBI database under accession numbers LC887936, LC887937, and LC887938 (RNA1, RNA2 and RNA3 of CMV-Pa3) and LC765220, LC765221, and LC765222 (RNA1, RNA2, and RNA3 of CMV-Rs1).

## Supplementary Materials

The following supporting information can be downloaded at: https://www.mdpi.com/article/10.3390/ijms262311755/s1.
