# Supplementary material for: A Single Amino Acid in Cucumber Mosaic Virus Determines Systemic Infection in Legumes: Species-Specific Differences in Key Residue Locations
Source: Int J Mol Sci. 2025 Dec 4;26(23):11755. doi: 10.3390/ijms262311755 (PMC12692615; doi:10.3390/ijms262311755)
Supplement: Supplementary file 1 [file ijms-26-11755-s001.zip › ijms-4002589-supplementary.pdf]

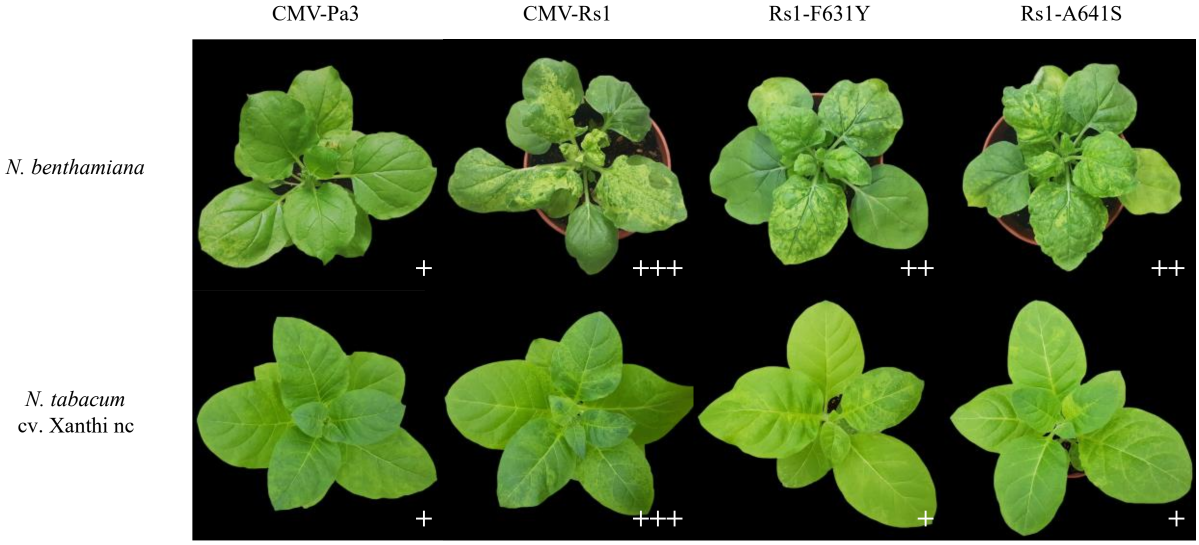

Supplementary Figure S1. Infectivity of CMV-Pa3, CMV-Rs1, and the point mutants (Rs1-F631Y and Rs1-A641S) in *Nicotiana benthamiana* and *N. tabacum* cv. Xanthi nc plants. Photographs of *N. benthamiana* and *N. tabacum* were taken at 12 and 14 dpi, respectively. Plus signs indicate the severity of symptoms.

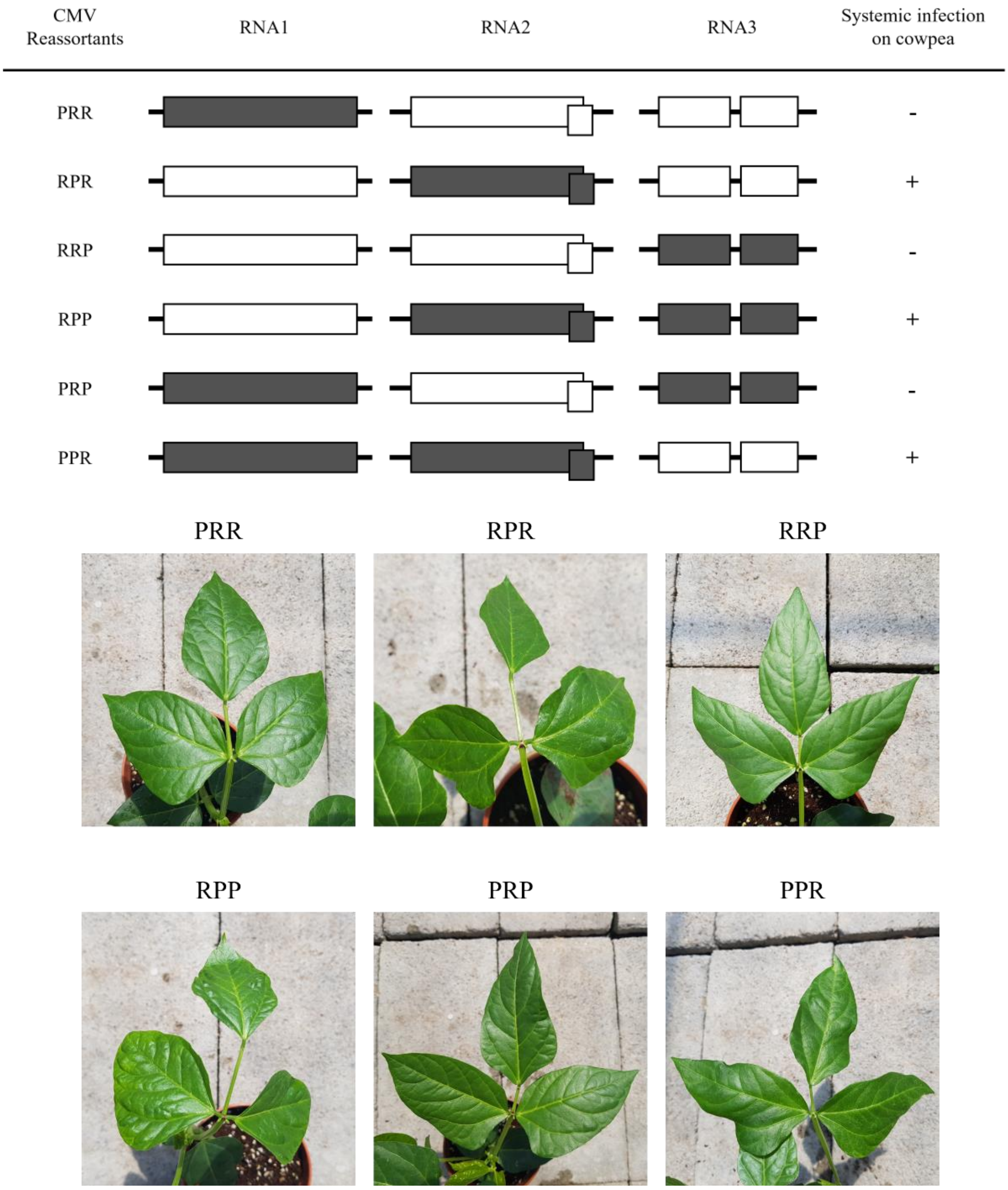

Supplementary Figure S2. Genome schematic of chimeras between CMV-Pa3 and CMV-Rs1, and the infectivity in cowpeas. RNA transcribed from CMV cDNA clones was inoculated into the cotyledons of cowpea plants. Symptoms were observed on the upper leaves 10 days after inoculation. Systemic infection was indicated as +(positive) or −(negative).

Supplementary Table S1. List of primers and sequence information used in this study

Primer	Sequence (5’ to 3’)
CMV-I-RNA1-*T7*-*Bam*HI	GCGGATCC*TAATACGACTCACTATA*GTTTATTTACARGAGCATACG
CMV-I-RNA2-*T7*-*Bam*HI	GCGGATCC*TAATACGACTCACTATA*GTTTATTYWCAAGAGCGTA
CMV-I-RNA3-*T7*-*Bam*HI	CGGGATCC*TAATACGACTCACTATA*GGTAATCTTACCACTGTGTGTG
CMV-R2-1580-F*	TTCATCCCTTGAGATGGCAGG
SSVK3-*Sph*I-R*	GCCTGCA*GCATGC*TGGTCTCCTTTGGAAGCCC
*Nco*I-Pa3-1851-F	ACCATGGCTGAGTTTGCCTG
*Pst*I-Pa3-2008-R	CTGCAGGCTCCATCACCTTAG
G1978P-F	CACAACTCTTTACAACATGGAAG
G1978P-R	CTTCCATGTTGTAAAGAGTTGTG
G2007P-F	TGATGGAACCTTCAGTACCATAT
G2007P-R	ATATGGTACTGAAGGTTCCATCA
